# Fractures of the Lower Extremity after E-Bike, Bicycle, and Motorcycle Accidents: A Retrospective Cohort Study of 624 Patients

**DOI:** 10.3390/ijerph20043162

**Published:** 2023-02-10

**Authors:** Thomas Rauer, Andrin Aschwanden, Benjamin B. Rothrauff, Hans-Christoph Pape, Julian Scherer

**Affiliations:** 1 Department of Traumatology, University Hospital of Zurich, Raemistrasse 100, 8091 Zürich, Switzerland; 2 Faculty of Medicine, University of Zurich, 8006 Zürich, Switzerland; 3 Department of Orthopaedic Surgery, University of Pittsburgh Medical Center, Pittsburgh, PA 15213, USA; 4 Orthopaedic Research Unit, University of Cape Town, H49 Old Main Building, Observatory, Cape Town 7700, South Africa

**Keywords:** polytrauma, road traffic accident, e-bike, lower extremity, injury pattern distribution

## Abstract

Electric bicycles (e-bikes) have gained enormous popularity in recent years, and as a result, they have successively become more involved in traffic accidents. The aim of the present study was to assess differences in severity and localization of injuries to the lower extremities after accidents with e-bikes, conventional bicycles, and motorcycles. A retrospective cohort-analysis of patients who sustained traumatic accidents with two-wheeled vehicles transferred to a level 1 trauma center in Switzerland was performed. We assessed patient demographics, injury pattern, and trauma severity (ISS), with a subgroup analysis of outcomes stratified by vehicle. In total, 624 patients (71% male) with injuries to the lower extremities after bicycle (*n* = 279), electric bike (*n* = 19), and motorcycle (*n* = 326) accident were included. The mean age of all assessed patients was 42.4 years (SD 15.8), with a significantly higher age in the e-bike cohort (*p* = 0.0001). High-velocity injuries were found significantly more often in the motorcycle and e-bike group. The motorcycle group had a significantly higher mean ISS (17.6) than the other groups (*p* = 0.0001). E-bike accidents produce a different injury profile to the lower extremities compared to motorcycle or bicycle accidents. Higher age, higher velocity, and different protective equipment seem to have an impact on these fracture patterns.

## 1. Introduction

E-bikes are touted as an environmentally friendly alternative to other means of transport and have become very popular in recent years. Furthermore, they have been proposed as a clean alternative to cars and to increase physical activity [1,2]. According to the motor assistance, e-bikes can be separated into two different groups based upon maximum speed (25 km/h or 45 km/h), and elderly individuals especially can make use of an efficient and economical fast mode of transport [3]. In 2018, 47.6 million e-bikes were sold globally, and sales continue to increase [4]. With an increasing number of e-bikes on public streets, the incidence of e-bike-related traumatic injuries has also risen [5]. A recent study by our study group showed that the injury pattern of e-bike accidents is more similar to bicycle accidents than motorcycle accidents [6]. Other studies have shown similar results, reporting increased injury severity and hospitalization rates and times, respectively, in e-bike accidents compared to conventional bicycle accidents [7,8,9]. However, data on specific body regions (e.g., lower extremities), after traumatic injury with two-wheeled vehicles are lacking. Based on the expected difference in speeds among the three groups of vehicles, we expect a difference in lower extremity injury severity. A graduation of injury severity is assumed, where we expect the most severe injuries in the motorcycle group, followed by the e-bike group, and finally the bicycle group. Thus, the aim of this study was to assess differences in the severity and location of injuries to the lower extremities after accidents with bicycles, electric bicycles, and motorcycles as part of a subgroup analysis of the aforementioned study [6].

## 2. Materials and Methods

This study was designed as a single-center retrospective cohort study and was approved by the cantonal ethic commission Zurich (PB_2016-01888). Informed consent was obtained from all subjects. It follows the Strengthening the Reporting of Observational Studies in Epidemiology (STROBE) guidelines for reporting observational studies [10].

### 2.1. Patients and Setting

This study included patients treated for a traffic accident at a level 1 academic trauma center between 2009 and 2018. All medical data were collected from electronic medical records during hospitalization and were retrospectively analyzed. Patients were followed until hospital discharge.

Patients were included in this study if they were treated after a traffic accident that involved an e-bike, bicycle, or motorcycle. Further details on patient recruitment and inclusion and exclusion criteria of the baseline population have been published previously [6].

Out of the initial cohort (*n* = 1796), only patients with fractures of the lower limbs were included. Patients were classified into one of three cohorts (e-bike, bicycle, and motorcycle) according to the involved vehicle during the traffic accident. Fracture classification was performed according to the AO-classification system [11]. We subsumed as distal tibia and distal fibula fractures all extra-articular fractures of the distal third of the lower leg, including 43-A fractures, according to the AO-classification system. We subsumed all fractures of the malleoli and the 43-B/C fractures, according to the AO-classification system, as ankle fractures. Open fractures were further classified according to the Gustilo–Anderson classification system [12]. In order to allow comparison of the general trauma severity, the injury severity score (ISS) was calculated. To compare the local severity of injuries, the maximum Abbreviated Injury Score (mAIS) was assessed [13,14,15].

### 2.2. Statistical Analysis

Statistical analysis was performed with the use of SPSS^®^ Statistics Desktop 26.0 for Mac (SPSS, Chicago, IL, USA). Data are presented as frequencies (*n*) and means with the standard deviation (SD). To assess differences in categorical data between the groups, a Chi-Square (Fisher’s exact test if applicable) test was used. To assess differences in continuous data, the non-parametric median or students’ t-test was used. The level of statistical significance was set at *p* < 0.05.

## 3. Results

### 3.1. Demographics

Of 1796 patients who sustained trauma in an accident with a two-wheeled vehicle, 624 patients had an injury to the lower extremities. Injuries to the lower extremities included fractures, contusions, and dislocations. The mean age of all included patients was 42.4 years (SD 15.8), and 21.8% were female.

The patients within the motorcycle group (mean 40.4 years, SD 15.4, *p* = 0.0001, range 16 to 89 years) and within the bicycle group (mean 43.8 years, SD 15.9, *p* = 0.022, range 16 to 85 years) were significantly younger than the patients within the e-bike group (mean 54.9 years, SD 12.6, range 26 to 75 years). The majority of the assessed patients were male (78%). In the bicycle as well as in the motorcycle cohort (71% and 87% versus 37%), a significant male predominance was seen (*p* = 0.0001).

### 3.2. Fracture Distribution

**Figure 1:**
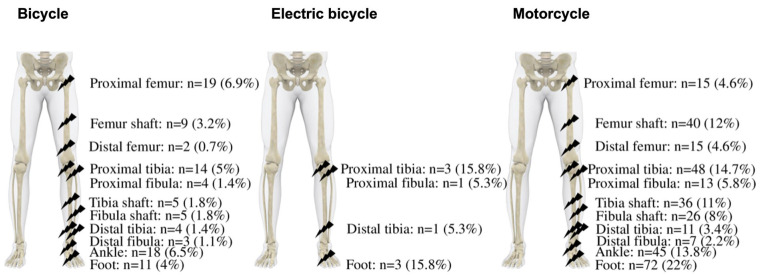
Distribution of fractures stratified by vehicle. Total numbers of depicted fractures and percentages (%) of total fracture number in each vehicle group.

According to the anatomical location, the distribution and severity of fractures in the three groups was as follows (Table 1, Figure 1). It must be noted that not all the assessed patients suffered from fractures, but sustained contusions or dislocations.

#### 3.2.1. Femur

In the bicycle group, 6.9% (N = 19) of fractures involved the proximal femur, whereas no patient in the e-bike group suffered from this injury. Fifteen patients from the motorcycle group had injuries of the proximal femur (4.6%). No difference between male and female patients was seen (*p* = 0.120 bicycle and *p* = 0.313 motorcycle).

The highest incidence of fracture to the femur shaft was found in the motorcycle group (12%, N = 40), followed by the bicycle group (3.2%, N = 9) (*p* = 0.004). No fractures of the femur shaft occurred in the e-bike group. No difference in terms of gender was seen in the bicycle group (*p* = 0.539) and in the motorcycle group (*p* = 0.392).

In addition, no fracture to the distal femur occurred in the e-bike group, whereas 15 patients (4.6%) in the motorcycle group and 2 patients (0.7%) in the bicycle group suffered an injury to the distal femur. Furthermore, no gender differences were found (bicycle *p* = 0.656 and motorcycle *p* = 0.748).

#### 3.2.2. Patella

There were five patella fractures (3 B and 2 C) in the bicycle group (1.8%). No patella fractures occurred with e-bikes. Ten fractures (1 A, 4 B, and 5 C) occurred in the motorcycle group (3.1%). No significant differences between female and male patients were observed (*p* = 0.676 and 0.436).

#### 3.2.3. Tibia

Significantly more fractures of the proximal tibia were observed in the motorcycle group (N = 48; 6 A, 21 B, and 21 C, 14.7%), followed by bicycle group (N = 14; 1 A, 10 B, and 3 C, 5.0%) compared to the e-bike group (N = 3; 3 C, 15.8%) (*p* = 0.0001). Significantly more female patients had an AO-B fracture (*p* = 0.021) in the bicycle group. In the motorcycle group, significantly more male patients suffered an AO-B or -C fractures (*p* = 0.046).

Regarding tibia shaft fractures, significantly more patients within the motorcycle group suffered from these injuries (N = 36; 11 A, 8 B, and 17 C, 11.0%) compared to bicycles (N = 5; 3 A, 0 B, and 2 C, 1.8%) and e-bikes (N = 0) (*p* = 0.001). No differences between male and female patients were found (*p* = 0.347 bicycle and *p* = 0.385 motorcycle). Significantly more distal tibia fractures occurred in the motorcycle group (N = 11; 2 A, 2 B, and 7 C, 3.3%) than in the bicycle group (N = 4; 3 B and 1 C, 1.4%) and the e-bike group (N = 1, 5.3%) (*p* = 0.019).

#### 3.2.4. Fibula

There was no significant difference between the motorcycle group (N = 13; 6 A and 7 B, 5.8%), the electric bicycle group (N = 1; 1 A, 5.3%), and the bicycle group (N = 4; 3 A and 1 B, 1.4%) regarding fractures of the proximal fibula (*p* = 0.178). There were also no differences between male and female patients within the groups (bicycle *p* = 0.296; motorcycle *p* = 0.436). Significantly more motorcyclists sustained fractures of the fibula shaft (N = 26; 13 A, 7 B, and 6 C, 7.9%) than bicyclists (N = 5; 4 A and 1 B, 1.8%) (*p* = 0.027). There was no gender-specific difference in the bicycle group (*p* = 0.796) as well as in the motorcycle group (*p* = 0.494). Regarding fractures of the distal fibula, we did not find any differences between the groups (*p* = 0.641). There were also no differences between male and female patients (bicycle *p* = 0.662, motorcycle *p* = 0.589)

#### 3.2.5. Ankle

Regarding fractures of the ankle, there was a significant predominance of motorcyclists (N = 45; 11 A, 28 B, and 6 C, 13.8%) compared to bicyclists (N = 18; 4 A, 8 B, and 6 C, 6.4%) (*p* = 0.029). There were no ankle fractures in the e-bike group. No difference between male and female patients was assessed within the bicycle group (*p* = 0.268) and the motorcycle group (*p* = 0.552).

#### 3.2.6. Foot

We did not distinguish foot fractures based upon region (i.e., forefoot, midfoot, and rearfoot). There were significantly more fractures of the foot in the motorcycle group (N = 72; 72 A, 22.1%) than in the bicycle group (N = 11; 11A, 3.9%) and e-bike group (N = 3; 3 A, 15.8%) (*p* = 0.0001). There was no gender-difference in the group of bicyclists (*p* = 0.604), in the group of e-bicyclists (*p* = 0.149), and in the group of motorcyclists (*p* = 0.611).

#### 3.2.7. Open Fractures

**Figure 2:**
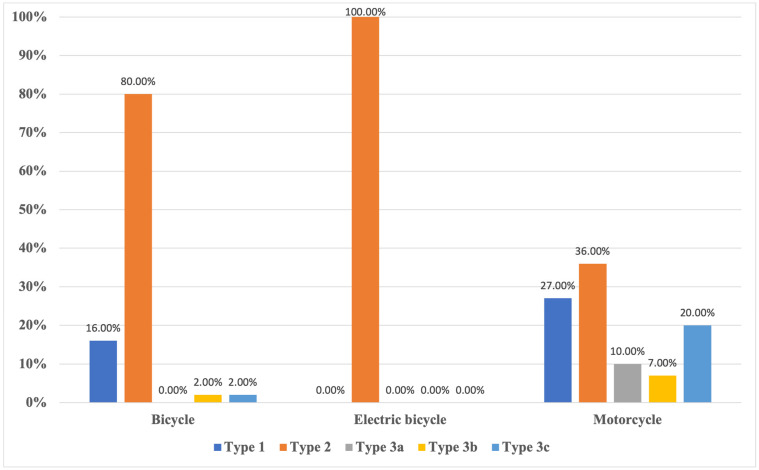
Distribution of open fractures stratified by vehicle group. Bars show percentages of the total number of open fractures classified according to the *Gustilo*—*Anderson Classification* (Type 1 to 3c).

In total, there were 159 open fractures of the lower extremities (25.5% of total fractures), as depicted on detail in Figure 2. Of these, 23.3% were type 1 according to the Gustilo–Anderson Classification (N = 37). Type 2 fractures were found in 50.9% of the cases. Type 3a fractures were found 10 times (6.3%) and type 3b fractures 9 times (5.9%). Type 3c fractures were found in 22 patients (13.8%). The motorcycle group contained the most open fractures (N = 107), followed by the bicycle group (N = 51) and e-bike group (N = 1) (*p* = 0.0001). Type 3 fractures mainly occurred in the motorcycle group (N = 39) (*p* = 0.001).

#### 3.2.8. Injury Pattern

Of all patients who sustained trauma to the lower extremities, 110 (17.6%) had a monotrauma of the lower extremity. Twenty-nine patients (4.6%) suffered from multiple injuries to the lower extremities. The majority of patients (N = 485, 77.7%) had polytrauma to the lower extremities and the upper body. There were no significant differences among the vehicle groups (*p* = 0.721) (Table 2). More female patients had multiple traumas to the lower extremities, but the difference did not reach statistical significance (8.1% versus 3.7%, *p* = 0.060).

#### 3.2.9. Injury Severity

**Figure 3:**
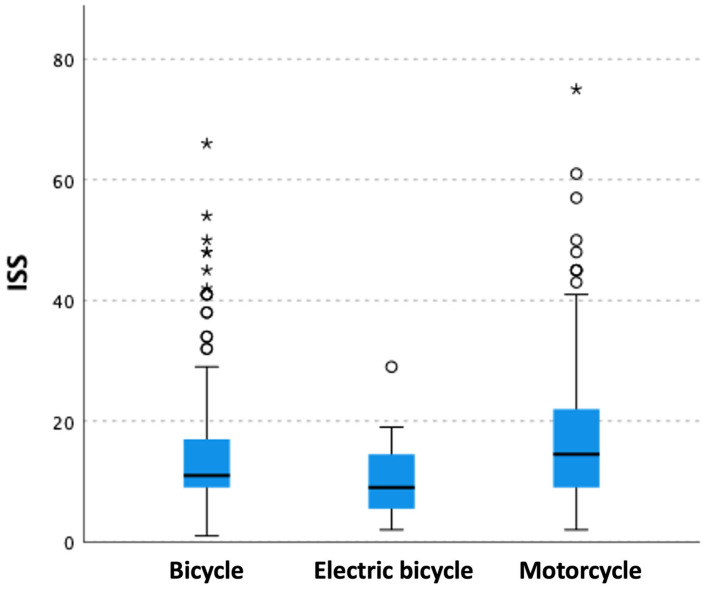
Mean Injury Severity Score (ISS) of patients stratified by vehicle. The boxplot shows mean and median ISS of patients stratified by their used vehicle. Circle = Outlier; Asterisks = Extreme Outlier.

Of 624 patients, we recorded the Injury Severity Score (ISS) in 500 patients (18.6% female). The mean ISS was 16.0 (range 1 to 75, SD 10.6) and 41% of patients had an ISS over 15. Almost half of the motorcyclists showed an ISS over 15 (N = 140; 49.6%), followed by bicyclists (N = 60; 30.2%) and electric bicyclists (N = 5; 26.3%) (*p* = 0.0001). The motorcycle group had a significantly higher mean ISS (17.63) than the bicycle group (14.09) and the e-bike group (14.09) (*p* = 0.0001) (Figure 3). Male patients showed a significantly higher mean ISS (16.6, SD 10.9) than their female counterparts (13.1, SD 9.0) (*p* = 0.004).

#### 3.2.10. Dislocations

We recorded six traumatic dislocations of the knee, which all occurred in the motorcycle group (*p* = 0.063). There were significantly more complete dislocations of the ankle joint–foot complex in the motorcycle group (N = 36), followed by the bicycle group (N = 7) and the e-bicycle group (N = 1) (*p* = 0.0001).

#### 3.2.11. Soft Tissue Injuries

**Figure 4:**
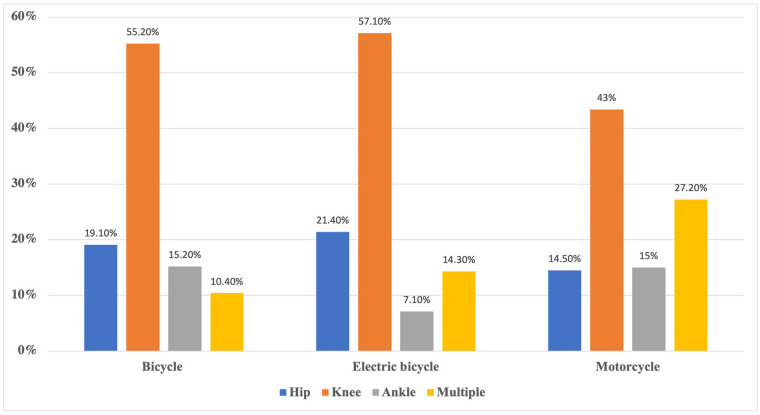
Distribution of soft tissue injuries/contusions stratified by vehicle. Bars show percentages of contusions to the hip, knee, ankle, or multiple locations amongst the total number of contusions stratified by the used vehicle.

In total, 417 patients suffered from soft tissue injuries (contusions). Bicyclists had significantly more soft tissue injuries (N = 230; 82.4%) than electric bicyclists (N = 14; 73.7%) and motorcyclists (N = 173; 53%) (*p* = 0.001). The distribution of contusions are depicted in Figure 4.

## 4. Discussion

The main objective of this study was to characterize the incidence and patterns of lower extremity injuries in e-bike accidents as compared to bicycle and motorcycle accidents.

Our study found the following main results:

1. The majority of patients had a combination of injuries to the lower extremities and upper body. Isolated injuries to the lower extremities were significantly less common.
2. E-bike injuries were limited exclusively to joint injuries of the lower leg, apart from foot injuries. The motorcyclists showed an injury pattern expected in a severe motor vehicle trauma, extending over the entire lower extremity. The group of cyclists showed fewer shaft fractures.
3. E-bicyclists who sustained lower limb injuries were older than bicycle or motorcycle riders.

We assessed the injuries of 624 patients who sustained trauma to the lower extremities with bicycles, e-bikes, and motorcycles. Patients with fractures of the lower extremity who had e-bike accidents were significantly older than the other groups. This finding is consistent with our recently published overall cohort [6]. A possible explanation for this phenomenon is the decreasing reaction time in elderly patients as well as less control of the relatively higher velocity of e-bikes compared to conventional bicycles. A study which investigated electric bicycle injuries using a questionnaire identified that age was not a predictive factor for accidents [16]. However, the authors feel that a possible explanation for our data is that younger patients have less e-bike accidents that lead to hospitalization, and therefore, these patients were not captured in our study, which took place at a level 1 trauma center. We found a male predominance in the motorcycle and bicycle group, but not in the electric bicycle group. Interestingly, male sex was found to be a risk-factor for trauma in e-bike accidents, which is not supported by our data [16]. However, it was previously reported that male motorcyclists have worse injuries and are more often injured than female motorcyclists, which is consistent with our data [17,18]. In terms of bicycle accidents, previous studies have shown male predominance, which is consistent with the presented data [19]. In our study, motorcyclists sustained significantly more fractures to the femur and the knee (proximal tibia), and especially, more severe fractures (AO-C) than the other groups. In addition, more foot and ankle fractures were found in this group. This seems logical, since motorcycle accidents have naturally higher kinetics, and these patients are, therefore, at a higher risk of high-velocity injuries and major fractures. This phenomenon is also reflected by the significantly higher mean ISS in the group of motorcyclists [20]. Interestingly, the most often fractured bone in the group of electric bicyclists was the tibia, and here it was always an intra-articular fracture, which is consistent with the data from a previous study on orthopedic injuries in e-bike accidents [21]. Surprisingly, fractures to the femur were detected in the motorcycle and bicycle group, but not in the e-bike group. The reason for this remains unclear. One explanation could be the small sample size of the e-bike group. Future studies with a larger sample size should reevaluate these findings. Regarding the injury severity of the fractures, we found more open fractures in the motorcycle group, which is consistent with previous studies on the comparison of injuries between different road users [20].

The majority of our patients had combination injuries to both the lower extremities and upper body. No difference between the cohorts were found. Approaching statistical significance, female patients had more polytrauma to the lower extremities, which is consistent with previous studies on road accidents in Sweden [20]. A previous study on e-bike accidents showed that 6.4% of patients had an ISS greater than 15, whereas data from China showed an ISS greater than 15 in 32.9% of the cases [21,22]. Our data showed an ISS over 15 in 26% of the cases, which seems to be somewhat in between these previous studies. Hu et al. showed major trauma (ISS > 15) in 16.9% of the bicycle cases, which was almost doubled in our cohort (30.2%) [22]. The authors feel that this gap in polytraumatized patients may be due to the performance of the study at a level 1 trauma center, whereas the previous study was not entirely performed at a level 1 trauma center. The highest incidence of polytraumatized patients as well as the highest mean ISS was seen in the group of motorcyclists, which is consistent with data from previous studies [23,24]. Due to the high velocity character of motorcycle crashes, most of the joint dislocations occurred in the group of motorcyclists. In our study, bicyclists and e-bicyclists had significantly more contusions, but less fractures, than the motorcyclists. This seems logical, since these two groups have lower velocity accidents but at the same time wear significantly less protective equipment and, therefore, suffer more soft-tissue injuries than fractures. This is in keeping with previous studies, which have shown the potential of mandatory protective gear to decrease injury severity in e-bike and commercial bicycle accidents [25,26].

Our results are consistent with current Swiss legislation, which requires only riders of “fast” e-bikes to wear a bicycle helmet, but no other protective gear. When it comes to motorcyclists, the current legislation dictates a special motorcycle helmet but does not require other protective gear. However, proper protective gear for motorcyclists is strongly advised, since not wearing protective gear in an accident can lead to decreased health insurance benefits and payments. Therefore, we believe that it can be assumed that most of the motorcyclists are wearing full-body protective gear [27].

Higher age, higher velocity, and different legislations and uses of protective equipment could have an impact on injury patterns. E-bike riders, who are often elderly patients, may benefit from adding protective gear to the lower extremities (over knee) and instruction courses, like motorcyclists. Further (prospective) studies with a larger sample size, recording worn protective gear, are needed to evaluate the benefit of protective gear in electric bicycle accidents.

### Strengths and Limitations

Since data was collected in a level 1 trauma center, the presented data reflects a broad spectrum of patients including urban as well as patients from rural areas. Furthermore, the included patients possessed a large range of injury severity. However, there are also some limitations. First, since data was collected from a level 1 trauma center, a higher level of trauma severity was likely present as compared to a lower acuity hospital, which can reflect a selection-bias. Furthermore, the group of electric bicycles only consisted of 19 patients, which is a rather small sample size and reflects limitations in terms of generalizability. In addition, we included only patients aged 16 years and older, so injury patterns in children remain unclear. Since this was a retrospective study, only the minority of patients had (incomplete) data on worn protective equipment. This should be recorded in further prospective multi-center studies from hospitals with different trauma levels to gain insights into difference in injury patterns in different healthcare settings.

## 5. Conclusions

E-bike injuries to the lower extremities differ from injuries sustained in motorcycle or bicycle accidents. In the e-biker group, tibia fractures, especially intra-articular tibia fractures, appear to occur more frequently. While isolated mono-injuries and multiple injuries to the lower extremities are much less common, the majority of injured e-bikers show combined injuries to both the lower extremities and the upper body.

## Figures and Tables

**Table 1:** Distribution of fractures stratified by vehicle. The table shows the total number of fractures stratified by the used vehicle. N shows the total of patients in each vehicle group. Chi-square test was used to determine differences between the vehicle groups. * Indicates statistical significance.

	Bicycle (*n* = 279)			N	Electric Bicycle (*n* = 19)			N	Motorcycle (*n* = 326)			N	*p*-Value
AO-Classification	A	B	C		A	B	C		A	B	C		
Proximal femur	11	8	0	19	0	0	0	0	14	1	0	15	0.088
Femur shaft	4	1	4	9	0	0	0	0	18	8	14	40	0.004 *
Distal femur	0	1	1	2	0	0	0	0	2	6	7	15	0.162
Patella	0	3	2	5	0	0	0	0	1	4	5	19	0.889
Proximal tibia	1	10	3	14	0	0	3	3	6	21	21	48	0.0001 *
Tibia shaft	3	0	2	5	0	0	0	0	11	8	17	36	0.001 *
Distal tibia	0	3	1	4	1	0	0	1	2	2	7	11	0.019 *
Proximal fibula	3	1	0	4	1	0	0	1	6	7	0	13	0.178
Fibula shaft	4	1	0	5	0	0	0	0	13	7	6	26	0.027 *
Distal fibula	2	1	0	3	0	0	0	0	2	5	0	7	0.641
Ankle	4	8	6	18	0	0	0	0	11	28	6	45	0.029 *
Foot	11	0	0	11	3	0	0	3	72	0	0	72	0.0001 *

**Table 2:** Injury pattern stratified by vehicle group.

	Bicycle (*n* = 279)	Electric Bicycle (*n* = 19)	Motorcycle (*n* = 326)
Single trauma lower extremities	52 (18.7%)	2 (10.5%)	56 (17.2%)
Multiple trauma lower extremities	10 (3.6%)	1 (5.3%)	18 (5.5%)
Polytrauma upper body and lower extremities	217 (77.7%)	16 (84.2%)	252 (77.3%)

## Data Availability

The raw data used in the analyses of this study are available in the authors’ database. Data is accessible on reasonable request.
